# No Difference in Mood and Quality of Life in DHEA-S Deficient Adults with Addison’s Disease vs. Type 2 Diabetes Patients with Normal DHEA-S Levels: Implications for Management of These Conditions

**DOI:** 10.3389/fpsyg.2017.00764

**Published:** 2017-05-11

**Authors:** Adrian H. Heald, Andreas Walther, Julian R. E. Davis, Gabriela Y. C. Moreno, John Kane, Mark Livingston, Helen L. Fowler

**Affiliations:** ^1^Department of Endocrinology, Salford Royal NHS Foundation Trust, University of ManchesterSalford, UK; ^2^Department of Psychology, TU DresdenDresden, Germany; ^3^Department of Endocrinology, Manchester Royal InfirmaryManchester, UK; ^4^Instituto Politecnico NacionalMexico City, Mexico; ^5^Clinical Biochemistry, Salford Royal NHS Foundation TrustSalford, UK; ^6^Department of Blood Sciences, Walsall Manor HospitalWalsall, UK; ^7^Behavioural Medicine, Salford Royal NHS Foundation TrustSalford, UK

**Keywords:** DHEA deficiency, Addison’s disease, mood, depression, quality of life, diabetes type 2

## Abstract

Patients with Addison’s disease have relatively high rates of depression and anxiety symptoms compared with population-based reference samples. Addison’s disease results in deficiency of dehydroepiandrosterone (DHEA) and DHEA-sulfate (DHEA-S). There is considerable debate about the specific effects of DHEA deficiency on energy level and mood. We measured emotional well-being in 16 patients with Addison’s disease and a group of 16 hospital attendees with type 2 diabetes. Participants completed the General Health Questionnaire-28 (GHQ-28), the Hospital Anxiety and Depression Scale (HADS), the World Health Organization’s quality of life assessment (WHOQOL-BREF) and the Holmes–Rahe life event scale. DHEA-S was low in Addison’s patients (Addison’s men: 0.5 ± 0.1 μmol/l [normal range: 2.1–10.8] compared with diabetes men: 3.2 ± 1.2 μmol/l; Addison’s women: 0.4 ± 0.01 μmol/l [normal range: 1.0–11.5] compared with diabetes women: 2.2 ± 0.71 μmol/l). Testosterone levels were similar in both groups studied. There were no differences in emotional well-being and quality of life (QOL) between patients with Addison’s disease and Type 2 Diabetes Mellitus as measured by GHQ-28 (Addison’s: 22.4 ± 2.6, Diabetes: 19.6 ± 2.7), HADS Depression (Addison’s: 5.4 ± 0.9, Diabetes: 4.5 ± 1.4), HADS Anxiety and WHOQOL-BREF. There were no gender differences in affective symptomatology within the Addison’s group. Life event scores were above average in both groups (Addison’s: 195 ± 39.6, Diabetes: 131 ± 43.8), but not significant for difference between groups as was GHQ-28 total score. Both groups scored highly on the GHQ-28 and the life event scale, indicative of poorer health perceptions than the general population. This could be due to the chronicity of both disorders. We have not identified any specific effects of DHEA-S deficiency on mood or QOL.

## Introduction

Addison’s disease is described as a clinical syndrome presenting with fatigue, anorexia, wasting, and hyperpigmentation. Addison’s disease results in deficiency of dehydroepiandrosterone (DHEA) and DHEA-sulfate (DHEA-S) in addition to glucocorticoids and mineralocorticoids (Arlt and Allolio, 2003). Replacement therapy with glucocorticoids and mineralcorticoids corrects most of the physiological problems. However, health related quality of life (QOL) may be impaired, with some individuals with Addison’s disease exhibiting significant anxiety and depressive symptoms (Fava et al., 1986; Lobo et al., 1988).

Løvås et al. (2002) compared the subjective health status of a large group of Addison’s patients to a population based reference sample. Despite the fact that many had been taking longstanding replacement therapy, they reported a reduced general health perception, decreased vitality and increased fatigue based on the evidence from the Short Form-36 and the Fatigue questionnaires. An explanation that has been proposed for the results reported by Løvås et al. (2002) is that the impaired QOL experienced by Addison’s patients is secondary to a reduction in the synthesis of DHEA and DHEA-sulfate (DHEA-S).

The context of the study is that a proportion of people with Addison’s disease report dysphoria as also do patients with type 2 diabetes. Both conditions have a profound impact on the individual’s QOL. However, the nature of the endocrine pathology is very different. We aimed here to look at whether the character of mood disturbance in Addison’s disease may be modulated by the phenomenon of low DHEA levels.

In healthy women, the synthesis of DHEA and DHEA-S occurs exclusively in the adrenal cortex. In men, it is estimated that 5% of DHEA-S and 10–25% of the circulating DHEA are secreted by the testes (Vermeulen, 1980). Once in the circulation DHEA-S can be metabolized back to DHEA by sulfohydrolases in peripheral and adrenal tissues. Bird et al. (1983) reported that 64 and 74% of the daily production of DHEA-S is converted to DHEA in women and men respectively, but only about 13% of DHEA is metabolized back to DHEA-S.

There is evidence that DHEA can act in the central nervous system (CNS) (Gurnell and Chatterjee, 2001). Concentrations of DHEA/DHEA-S are considerably higher in the brain than any other organs. DHEA can act on two receptors within the brain, the inhibitory GABA-benzodiazepine-receptor complex (GABA-RC) and the *N*-methyl-D-aspartate (NMDA) receptor, a major receptor for excitatory amino acids. Receptor studies suggest that these hormones have the potential to exert clinically relevant effects in the CNS (Kroboth et al., 1999).

Low basal DHEA levels were shown to be associated with worse mood and reduced vitality (Walther et al., 2016), while promising effects on well-being and libido have been reported after DHEA administration to adult women with adrenal insufficiency suggesting the importance of low androgen levels in these women (Arlt et al., 1999; Hunt et al., 2000). A significant improvement in self-esteem and mood and a decrease in fatigue were reported during DHEA treatment in both sexes (Walther and Ehlert, 2015). The changes in men were independent of circulating total testosterone, suggesting that DHEA has a direct central action (Achermann and Silverman, 2001). However, combined dysregulation of DHEA and testosterone secretion has been linked to mood disorders especially in men (Walther et al., 2017). Also a previous study has shown that DHEA therapy for 6 weeks has a significant beneficial effect on major depression (Wolkowitz et al., 1999).

In addition to affective changes associated with neurochemical imbalances, the impact of any chronic disease on daily life can be significant (White, 2001). For example compliance with medication, regular hospital visits and concern about future health may all negatively impact on emotional well-being and QOL. There is a wealth of published evidence that long term conditions such as diabetes are associated with a higher prevalence of mental health problems and impoverished QOL in multiple domains (Gonder-Frederick et al., 2002).

In view of this we compared a group of Addison’s patients (all confirmed to be DHEA deficient) with type 2 diabetes patients, free of major organ complications in order to look for any specific effects of DHEA deficiency on emotional well-being and QOL. A secondary goal was to explore gender differences in affective symptoms in Addison’s disease.

## Materials and Methods

### Study Sample

This was a group comparison study. Groups were defined by the diagnosis of Addison’s disease or well-controlled type 2 diabetes mellitus without major organ complications. The rationale for choosing the diabetes group for comparison was that like Addison’s disease patients they have an enduring metabolic disorder which requires regular monitoring and physician input, daily medication and if the patient does not take the prescribed medication they will become unwell. All patients attending the Endocrine Clinic at Hope Hospital, Salford and Manchester Royal Infirmary who had been diagnosed with Addison’s disease and who were aged between 18 and 79 years of age were invited by letter to participate in the study.

All participants in the Addison’s group had primary adrenal insufficiency and patients with secondary adrenal insufficiency were excluded. Type 2 diabetes patients of a similar age and gender distribution were invited to participate.

The Addison’s group inclusion criteria were: adults with a diagnosis of Addison’s disease confirmed by Short Synacthen test (250 μg of soluble Synacthen given intramuscularly with failure of 30 min cortisol to rise above 400 nmol/l), who had been diagnosed more than 1 year previously. The comparison subjects were patients with type 2 diabetes mellitus attending outpatient clinics at Hope Hospital. The inclusion criteria were the diagnosis of type 2 diabetes mellitus (Alberti and Zimmet, 1998) and diagnosis more than 1 year previously.

The exclusion criteria for both the Addison’s group and diabetes group were: diagnosed unrelated pre-morbid chronic mental illness or disabling physical illness, unrelated neurological disorder, and hearing, motor or language barriers that prevented the structured interview for being carried out. On the basis of these criteria nine potential participants were excluded from the study.

Ethical approval was obtained from Salford and Trafford Local Research Ethics Committee and Central Manchester Local Research Ethics. All subjects gave written informed consent in accordance with the Declaration of Helsinki.

### Medical Assessment

A detailed medical and drug history was obtained by casenote review. Thyroid replacement therapy was considered adequate if circulating total thyroxine was within the normal range. Steroid replacement was considered adequate if the patient was receiving between 15 and 30 mg of Hydrocortisone per day. The type 2 diabetes patients were on oral hypoglycemic medication and/or insulin. Height and weight were measured by one of the investigators (PA) and subsequently the body mass index (BMI = kg/m^2^) was calculated.

### Biochemical Analysis

A blood sample was taken in a clotted tube and sent promptly to the laboratory for storage at -70°C until analysis. Blood samples were sent to Hope Hospital, Salford for analysis.

Androstenedione (between run inter-assay coefficient of variation [CV] < 8.9%), sex hormone binding globulin (between run inter-assay CV < 10.1%) and DHEA-S (between run inter-assay CV < 11.0%) was analyzed by enzyme labeled immunoassay (DPC Immulite 2000; Los Angeles, CA, USA). Testosterone was measured using a chemiluminescent labeled immunoassay (between run inter-assay CV < 9.3%) (Abbott Architect analyser, Abbott Laboratories, Abbott Park, IL, USA). For some female samples the testosterone was measured after extraction of the sample into di-ethylether according to a standard protocol.

### Psychometric tests

At interview the patients were asked to answer a series of questionnaires listed below:

#### (1) The Hospital Anxiety and Depression Scale (HADS)

The Hospital Anxiety and Depression Scale (HADS) is used for detecting states of depression and anxiety in the setting of a hospital medical outpatient clinic (Zigmond and Snaith, 1983). It is a widely used measure of anxiety and depression in general medical populations with a higher score indicating greater symptomatology, has been validated with a UK population and is relatively brief (Herrmann, 1997; Crawford et al., 2001).

#### (2) World Health Organization’s Quality of Life Assessment (WHOQOL-BREF)

The World Health Organization’s quality of life Assessment (WHOQOL-BREF) is an abbreviated version of the WHOQOL-100 assessment. It produces scores for four domains related to QOL: physical health, psychological health, social relationships and environment. It also includes a facet on overall QOL and general health. The WHOQOL-BREF domain scores demonstrated good discriminating validity, content validity, internal consistency, and test and re-test reliability (Skevington et al., 2004).

#### (3) General Health Questionnaire-28 (GHQ-28)

The General Health Questionnaire-28 (GHQ-28) is used in consulting settings aimed at detecting those with a diagnosable psychiatric disorder and has been well-validated (Goldberg and Hillier, 1979). The 28-item GHQ consists of four subscales: somatic symptoms, anxiety and insomnia, social dysfunction, and severe depression.

#### (4) Visual Analog (Likert) Scale (VAS)

The VAS measured five separate domains (one scale for each): happiness, concentration, memory, energy, and anxiety. Each domain is rated out of 10 with “0” indicating the least happiest, the least energetic they have ever been and “10” reflecting the most happiest, or most energetic they have ever been.

#### (5) Holmes–Rahe Life Event Rating Scale

Holmes and Rahe suggested that major life events good or bad were potentially stressful. This scale measures the impact of different events. There are over forty events on the scale and people mark those which have affected them in the previous 12 months. High scores have been found to relate to an increased chance of physical or mental illness (Holmes and Rahe, 1967).

### Statistical Analysis

Analyses were carried out using the statistical package SPSS version 11.5. Analysis of variance was used to compare age, duration of illness, and BMI between the diagnostic groups. Mann–Whitney-*U* testing was used to compare means between the groups because of the small sample size, non-parametric distribution, and two independent groups. Level of significance was set at α = 0.05.

## Results

### Demographic and Treatment Characteristics of the Groups

As presented in **Table 1**, there were 32 participants in total: 16 patients with Addison’s disease (eight women and eight men) and 16 patients with type 2 diabetes (eight women and eight men). Their ages ranged between 35 and 79 years. Participants in the male diabetes group were older than the male Addison’s group but the ages of female participants were similar in the two groups. The majority of participants in both groups were between the ages of 45 and 65 years. BMI was higher in the male diabetes group than their respective Addisonian counterparts but similar in both female Addison’s and diabetes groups. 81% of Addison’s patients and 61% of type 2 diabetes patients approached agreed to participate in the study.

**Table 1:** Comparison of age, duration of illness, and body mass index, between the Addison’s patient group and type 2 diabetes group.

	Addison’s patient group (male)	Diabetes group (male)	Addison’s patient group (female)	Diabetes group (female)	Test for differences between the groups Addison’s vs. type 2 diabetes
Age in years Mean/SD (range)	54.8/12.4 (40–79)	58.5/8.6 (44–68)	48.4/10.0 (35–61)	48.5/8.6 (38–63)	Men *F* = 3.2, *p* = 0.01 Women *F* = 0.3, p NS
Duration of illness (years) Mean/SD (range)	16.3/8.7 (1.4–28)	8.7/5.0 (1.6–17)	11.6/6.4 (1.6–20)	11.6/6.0 (2.8–23)	Men *F* = 4.3, *p* = 0.003 Women *F* = 0.1, p NS
Body mass index (kg/m^2)^ Mean/SD (range)	25.2/3.9 (19–30.1)	33.1/7.9 (26–49)	30/5.9 (23–38.6)	28.4/2.0 (26–31)	Men *F* = 3.5, *p* = 0.009 Women *F* = 2.3, *p* = 0.01

All patients with Addison’s disease were taking hydrocortisone and fludrocortisone (except one, who was only taking hydrocortisone) replacement therapy as shown in **Table 2**. No patients were taking DHEA replacement therapy. Seven were taking thyroxine and antihypertensive drugs, 12 out of the 16 were on other treatment regimes.

**Table 2:** Description of medical treatment for the Addison’s patient group and the type 2 diabetes group.

Variable		Addison’s female	Addison’s male	Diabetes male	Diabetes female	Total
Fludrocortisone replacement	Yes No	7 1	8 0	0 8	0 8	15 17
Hydrocortisone replacement	Yes No	8 0	8 0	0 8	0 8	16 16
Thyroxine replacement	Yes No	4 4	3 5	0 8	0 8	7 25
DHEA replacement	No	8	8	8	8	32
Oral anti-diabetic drugs	Yes No	0 8	0 8	6 2	5 3	11 21
Insulin	Yes No	0 8	0 8	5 3	5 3	10 22
Antihypertensive drugs	Yes No	3 5	4 4	7 1	7 1	21 11
Other treatment	Yes No	6 2	6 2	8 0	5 3	25 7

Within the diabetes group no patients were taking hydrocortisone, fludrocortisone, DHEA replacement, or thyroxine. Seven patients were taking both oral anti-diabetic medication and insulin; of the remaining patients six were taking oral hypoglycemic agents alone and three were on insulin treatment alone. All but two were on antihypertensive treatment.

### Biochemical Variables in Addison’s and Type 2 Diabetes Groups

**FIGURE 1:**
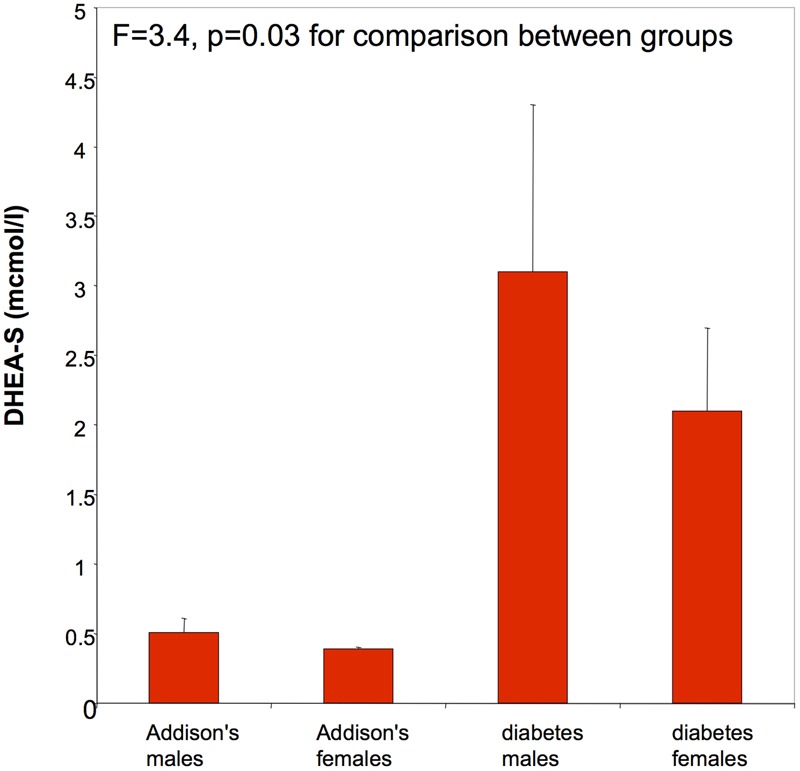
**DHEA-S (μmol/l) was low in male Addison’s patients (mean ± SE: 0.5 ± 0.1; normal range mean: 2.2–15.2) and female patients (mean ± SE: 0.4 ± 0.01; normal range mean: 1.0–12.0).** DHEA-S levels were normal in type 2 diabetes patients.

**FIGURE 2:**
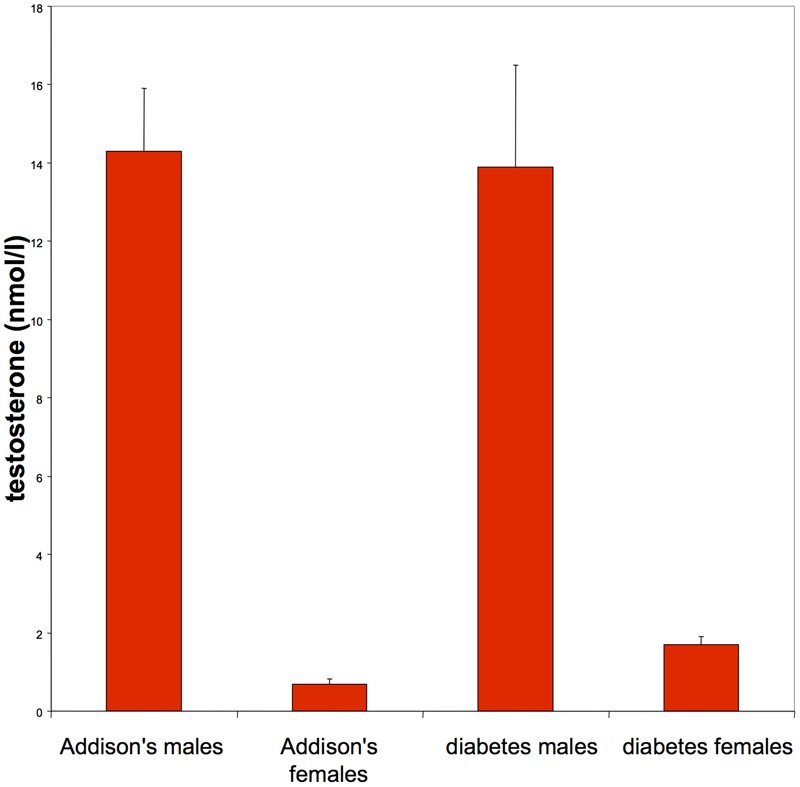
**Testosterone levels were normal in men (normal range mean: 10.0–35.0 nmol/l) and women (normal range mean: 0–2.4 nmol/l) in both Addison’s patients and type 2 diabetes patients**.

**Figure 1** shows that DHEA-S was low in Addison’s patients [men 0.5 ± 0.1 (mean ± SE) μmol/l (normal range 2.1–10.8), women 0.4 ± 0.01 μmol/l (1.0–11.5)] compared with diabetes men (3.2 ± 1.2 μmol/l) and diabetes women (2.2 ± 0.71.2 μmol/l). No type 2 diabetes patients had levels of DHEA-S below the laboratory normal range. Both male and female Addison’s and diabetes patients had normal testosterone (**Figure 2**) and SHBG levels [Addison’s men 28.0 ± 4.7 nmol/l (mean ± standard error of the mean), diabetes group men 34.8 ± 6.0 nmol/l: (normal range for men 13–71 nmol/l); Addison’s women 52.5 ± 8.9 nmol/l, diabetes group women 63.2 ± 10.7 nmol/l: (normal range for women 18–114)]. There were no significant differences in circulating testosterone and SHBG levels by gender between the Addison’s and diabetes groups.

### Differences in Mood and Quality of Life Scores between Male and Female Addison’s Patients

Each questionnaire was used to detect any differences in each patients QOL and general well-being. The Mann–Whitney-*U* statistical analysis showed no significant differences in any of the domains of QOL measured (domains listed in **Table 3**) between the male and female sex within the Addison’s patient group.

**Table 3:** Comparison between males and females within the Addison’s patient group for mood and quality of life.

	Addison’s males (Mean/*SD*)	Addison’s females (Mean/*SD*)	Normative data Mean (*SD*)	*P*
GHQ-28 (total)	22.4/2.6	19.6/2.7	16.1 (3.8)	0.18
HADS anxiety	7.1/1.9	6.4/0.6	6.1 (3.8)	0.2
HADS depression	5.4/0.9	4.5/1.4	≤3.7 (3.1)	0.2
WHO quality of life	4.1/0.3	4/0.2		0.68
WHO general health perception	3.1/0.4	3.1/0.4	*A higher score out*	0.95
WHO physical health	53.3/6.1	66.5/4.9	*of 100 reflects*	0.04
WHO psychological	61/5.7	72.8/5.8	*fewer problems*	0.16
WHO social relationships	63.3/6.4	75/9.6		0.43
WHO environment	72.9/6.6	78.4/5.1		0.59
VAS happiness	6.3/0.7	6.4/1.3	0 worst – 10 best	0.5
VAS concentration	5.4/0.6	7/0.9	0 worst – 10 best	0.1
VAS memory	5/0.8	5/0.8	0 worst – 10 best	0.9
VAS energy	4.8/0.8	5.5/0.7	0 worst – 10 best	0.3
VAS nervous	7.6/0.8	7.8/0.7	0 worst – 10 best	0.9
Social readjustment scale (life events)	195/39.6	131/43.8	*150 or less*	0.1

*GHQ-28, General Health Questionnaire-28; HADS, Hospital Anxiety and Depression Scale; WHO, World Health Organization; VAS, Visual Analog Scale.*

Each group had above average GHQ-28 scores but not HADS depression scores when compared to normative data (Crawford et al., 2001), which is consistent with previous studies demonstrating increased risk of mental health problems with long term medical conditions. However, neither group exhibited clinically significant levels of depressive or anxiety related symptomatology with no significant gender differences in Addison’s patients or those with type 2 diabetes. The WHOQOL results suggested that both men and women with Addisons’ reported at least average QOL, and consistent with this, their visual analog rating averaged between 50 and 80%, which implies they are modestly content in all those rated areas.

### Comparison of the Addison’s Patient Group and the Type 2 Diabetes Group for Mood and Quality of Life

Each questionnaire was used to detect differences between study groups. The Mann–Whitney-*U* statistical analysis displayed no differences in their perception of QOL, general well-being, anxiety, depression, health, personal psychological state, social relationships, the environment, happiness, concentration, memory, energy, and recent life events as presented in **Table 4**.

**Table 4:** Comparison of the Addison’s patient group and the type 2 diabetes patient group.

Variable	Addison’s group (Mean/SD)	Type 2 diabetes group (Mean/*SD*)	Normal range Mean (*SD*)	*P*
GHQ-28 (total)	21/1.8	21/2.3	16.1 (3.8)	0.7
HADS anxiety	6.75/0.9	6.2/0.9	6.1 (3.8)	0.7
HADS depression	4.9/0.8	5/0.7	3.7 (3.1)	0.8
WHO physical health	59.9/4.2	52.5/5.4	*A higher score*	0.3
WHO psychological	66.9/4.2	63.5/2.9	*out of 100*	0.4
WHO social relationships	69.1/5.8	71.2/4.0	*reflects fewer*	0.8
WHO environment	75.6/4.1	71.9/3.7	*problems*	0.6
VAS happiness	6.5/0.5	7.5/0.6	0 worst – 10 best	0.16
VAS concentration	6.2/0.6	7.0/0.7	0 worst – 10 best	0.3
VAS memory	5.0/0.5	5.2/0.5	0 worst – 10 best	0.7
VAS energy	5.1/0.5	5.1/0.5	0 worst – 10 best	0.9
VAS nervousness	7.7/0.5	8.8/0.32	0 worst – 10 best	0.2
Social readjustment scale	162.8/29.7	166.5/29.0	≤150	0.7

*GHQ-28, General Health Questionnaire-28; HADS, Hospital Anxiety and Depression Scale; WHO, World Health Organization; VAS, Visual Analog Scale.*

Both diabetes and Addison’s groups, had above average GHQ-28 scores, which is consistent with previous studies demonstrating increased risk of mental health problems with long term medical conditions. However, neither group exhibited clinically significant levels of depressive or anxiety related symptomatology. The WHOQOL results suggested that both groups reported at least average QOL, and consistent with this, their visual analog ratings ranged between 50 and 88% (on or above the median).

### Life Event Scores (Holmes–Rahe Social Readjustment Rating Scale)

**FIGURE 3:**
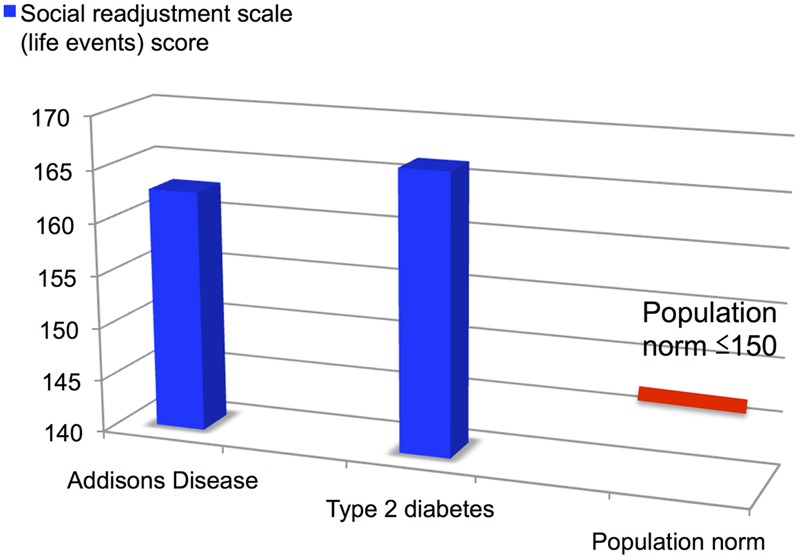
**Social readjustment scale life events scores for Addison’s disease and Type 2 diabetes patients vs. population normative range**.

Life event scores were slightly above average in both groups (see **Figure 3**): Addison’s 162.8 ± 29.7, Diabetes 166.5 ± 29.0 (score of 150 points or less indicates a relatively low amount of life change and a low susceptibility to stress-induced health breakdown), pNS for difference between groups. This is indicative of a greater number of independent life events than average for both groups.

## Discussion

The aims of this study were to assess differences in emotional well-being and QOL in Addison’s disease that could be related specifically to DHEA-S deficiency, and to explore any gender differences in affective symptomatology in patients with Addison’s disease. Our results show that there were no differences in emotional well-being and QOL between patients with Addison’s disease and type 2 diabetes mellitus, although GHQ-28 scores were higher than normal in both groups. They also confirm there were no gender differences in affective symptomatology within the Addison’s group.

The findings of this current study are important in the context of previous research. Hunt et al. (2000) found an improvement in mood and fatigue after DHEA replacement in Addison’s disease in a randomized double blind trial. Arlt et al. (1999) and Achermann and Silverman (2001) found improvements in well-being and libido after DHEA administration. Our study suggests that there are no significant differences between the Addison’s and type 2 diabetes patients in the domains assessed, although GHQ-28 scores were not normal for either group. We did not carry out any specific assessment of libido, which has been shown to be lower in women with Addison’s disease (Arlt, 2002), so any conclusions drawn do not relate specifically to this.

Schmidt et al. (2005) have described a reduction in HADS rating score and improved libido with DHEA replacement. We are not arguing against the use of DHEA as an agent which has the potential to improve mood, energy levels and libido among other psychological variables in an appropriate clinical setting but rather that the decrement in these areas seen in Addison’s disease may not be specifically associated with the endocrine abnormalities seen in this disorder.

Kroboth et al. (1999) reported that DHEA-S concentrations are higher in males than in females. In our study, DHEA-S levels were toward the lower end of the normal range in men and women in the type 2 diabetes patient group. Kroboth et al. (1999) reported that an insulin infusion acutely lowers serum DHEA and DHEA-S concentrations, increases the metabolic clearance rate of DHEA and inhibits adrenal androgen production. Thus insulin treatment may be a factor in the diabetes group having a lower level of DHEA-S concentration than in a population not taking insulin.

Participants with Addison’s and diabetes were not as closely matched for age as initially intended. This variation in clinical characteristics may have influenced the results. As the diabetes group were generally older than the Addison’s group, they may have problems that are age related and could contribute to the complications of their disorder. This could be a confounding factor in their perception of their general well-being and QOL, given that DHEA levels are known to fall with age in both sexes (Parker et al., 2000). However, both groups reported at least average QOL (WHOQOL-BREF assessment).

Even though there were no obvious differences between the two groups, both groups scored highly on the GHQ-28, which suggests an increased risk of mental health problems than in the general population. This could be due to the psychological impact of long-term physical illnesses and the associated complications. The slightly elevated independent life events than average for both groups is intriguing and may indicate an association between chronic endocrine disorders and an increased incidence of adverse life events. This may be consistent with previous evidence of increased psychosocial problems with diabetes (Gonder-Frederick et al., 2002).

An important observation of the investigators was that the type 2 diabetes patients, were more reluctant to participate than the Addison’s group. The continual need of self assessment and regulation, though essential, must be tiresome and maybe responsible for the apathy of the diabetes group (White, 2001).

Five questionnaires were used to assess the QOL and social functioning of both groups. The HADS and GHQ-28 scales are reliable instruments for screening for clinically significant anxiety and depression in patients attending general outpatients clinic. Although Zigmund and Snaith’s original conception of the HADS as measuring independent dimensions of anxiety and depression has been found untenable, it is legitimate to obtain a score on the total scale if a measure of general psychological distress is required (Crawford et al., 2001). The social readjustment rating scale is limited as the scale does not allow for individual differences in reactions to events, some even criticize it for putting positive and negative events together. This existing scale cannot be used to determine the role of varying types of life changes (favorable or adverse) in the occurrence of illness. Other problems include ambiguity of items, the confounding of independent and dependent variables, and lack of item specification (Mechanic, 1975).

### Limitations

When interpreting these results some limitations need to be considered. The power of the study is low due to small sample size. Thus, due to the low number of participants it would only able to detect large effect sizes. Furthermore, there were differences in age between the diabetes and Addison’s men inherent in the way that recruitment was carried out. The invitation to participate and recruitment was carried out with consecutive attendees at outpatient clinics at Salford Royal Hospital. Nevertheless, the purpose of the study was to test the hypothesis that did DHEA-S deficiency did affect mood and QOL in Addison’s disease in comparison with another group with a long-term life altering condition. Another type of control group for example type 1 diabetes patients might have been chosen. We do feel that the choice of type 2 diabetes patients is however, valid as discussed before.

## Conclusion

This study was constructed to investigate any changes in general well-being and QOL among patients with Addison’s disease. Even though there were no obvious differences between the two groups, the GHQ-28 results implied that both groups scored highly and may have poorer perceptions concerning their general health, than the population. This could be due to the chronicity of both disorders and the complications that accompany them. We have in this study not identified any specific effects of DHEA-S deficiency on mood or QOL.

## Author Contributions

AH, JD, JK, and HF designed and conducted the study. ML performed the blood analyses. AH and HF wrote the first draft of the manuscript. JD, JK, GM, and ML critically reviewed the manuscript. AH and HF subsequently reviewed further editing from the coauthors. AW critically reviewed the final manuscript and edited it to its final form.

## Conflict of Interest Statement

The authors declare that the research was conducted in the absence of any commercial or financial relationships that could be construed as a potential conflict of interest.
